# Knowledge of malaria control and attitudes towards community involvement among female community volunteers: effect of capacity building in a rural community, Southeast Nigeria

**DOI:** 10.11604/pamj.2021.39.151.25685

**Published:** 2021-06-28

**Authors:** Adaoha Pearl Agu, Chukwuma David Umeokonkwo, Nelson Chibueze Eze, Christian Obasi Akpa, Richard Chukwuka Nnabu, Ifeyinwa Chizoba Akamike, Ijeoma Nina Okedo-Alex, Chihurumnanya Alo, Jesse Chigozie Uneke

**Affiliations:** 1 African Institute for Health Policy and Health Systems Ebonyi State University Abakaliki, Abakaliki, Nigeria,; 2 Department of Community Medicine, Ebonyi State University Abakaliki, Ebonyi State, Nigeria,; 3 Department of Community Medicine, Alex Ekwueme Federal University Teaching Hospital Abakaliki, Ebonyi State, Nigeria,; 4 National Malaria Elimination Program Department of Public Health, Federal Ministry of Health Abuja, Nigeria,; 5 Department of Microbiology, Ebonyi State University, Abakaliki, Nigeria

**Keywords:** Malaria, community volunteers, capacity building, Ebonyi

## Abstract

**Introduction:**

community volunteers have limited skills but are an important link between the community and health facilities. We determined the effect of a capacity building intervention on knowledge of malaria control and attitudes towards community involvement among female community volunteers as part of a larger community-based intervention study on pregnant women and children under five.

**Methods:**

we conducted a before and after intervention study (no randomization or controls) among female community volunteers in Amagu community in Abakaliki Local Government Area. The intervention consisted of training sessions on knowledge of malaria and its control. The training took the form of lectures, role plays and practical demonstrations. Supportive supervision by trained community health extension workers was also provided during their field work. We compared pre-training test and post-training test scores after six months interval and analysed the data using paired t test at 5% level of significance with EPI INFO software version 7.2.3.

**Results:**

the mean age of the participants was 28.5(± 6.0) years. All had a minimum level of secondary education. There was significant improvement in the mean scores of their knowledge of malaria signs and symptoms (p < 0.001), preventive measures (p < 0.001) and appropriate drug treatment (p < 0.001) in the post-training test when compared with the pre-training test. The overall mean knowledge scores pre and posttest were 147.8 and 169.8 respectively (p < 0.001) out of a maximum achievable score of 195. Also there was significant improvement in the perception of the participants on community involvement in promoting referral of pregnant women with fever (p = 0.001), the use of intermittent preventive therapy with sulphadoxine-pyrimethamine (p = 0.048) and funding initiatives to sustain activities (p = 0.037).

**Conclusion:**

capacity building of female community volunteers coupled with supportive supervision by trained community health workers improved the female community volunteers´ knowledge of malaria, its control and their perception of community involvement in control activities. It is recommended that the use of community volunteers as a low cost health resource can be explored further for incorporation into existing policies on malaria control in resource constrained environments.

## Introduction

Combatting malaria is a continuing concern for World Health Organization (WHO) and the Roll Back Malaria partnership to End Malaria [1] and indeed a major area of interest within the field of public health currently. Despite concerted efforts at the control of malaria, the WHO AFRO Region, where 92% of the 219 million cases and 93% of the 435,000 deaths globally have occurred, bears the greatest burden [1]. In Nigeria where 25% of all cases and 19% of all deaths attributed to malaria globally occur [1], malaria contributes 11% to the maternal mortality and 30% to the under-five mortality [2]. The National Demographic Health Survey 2018 report [3] reveals that a large proportion of the vulnerable population at risk of malaria- pregnant women and under five children - are unprotected in Ebonyi State. Only 24.2% of women aged 15-49 years received three or more doses of Intermittent Preventive Treatment with Sulfadoxine- Pyrimethamine (IPTp) during the pregnancy resulting in the most recent birth. Similarly only 17% of the under five children who had had fever in the two weeks preceding the survey had their blood tested for malaria (South East Nigeria figure) [3]. Early diagnosis and appropriate treatment of malaria especially at village level has been identified as part of the comprehensive approach needed to control malaria [1]. However, one of the greatest challenges to achieving this has been the observed care seeking pattern for treatment of malaria/ febrile illness especially prevalent among lower socioeconomic groups where the preferred first providers are patent medicine vendors where the treatment given is most probably inappropriate [4-7]. According to the NMIS 2015 [2], the first place of treatment for children under five years with fever, sought by 57% of household members, was pharmacies, chemist stores and patent medicine vendors [2].

The promotion of desirable behavior for malaria prevention and treatment at the community, household and individual levels is in line with the objectives of the National Malaria Strategic Plan 2014-2020 [8]. The use of promotional interventions is likely to achieve this objective. Promotion of appropriate care seeking by both trained community health workers and community health workers with limited training is an evidenced based integrated MNCH intervention and delivery strategy reported in previous studies [9]. Apart from trained community health workers who have been recognized as an integral resource in health systems in resource poor settings [10-12] and their promotion by WHO for Integrated Management of Childhood Illnesses (IMCI) [13] the use of community volunteers as another low cost health resource is gaining increasing attention especially in provision of health education and referral services in the community [14-17] and even in implementing public health programs such as family planning, immunization and safe motherhood in Nepal [18] and management of childhood illness in Bangladesh [19]. Community health volunteers have limited skill as they are not the formally trained community health workers but have been reported as key agents in malaria control [20] and are an important link between the community and health facilities. It has been suggested that strengthening the role of community volunteers in PMTCT interventions can be done through improving their training with the use of appropriate educational materials and support of required resources [21]. Supervision and training were found from a systematic review to be facilitating factors for a better community health worker performance [22]. Community health volunteers in Nigeria are used in areas such as health promotion for breastfeeding, mental health education campaigns, health education and net distribution for malaria [23, 24]. There is little information on the effect of capacity building of community health volunteers in South East Nigeria on their knowledge of and attitudes towards malaria control specifically and diseases generally. This study aimed to determine the effect of a capacity building intervention on knowledge of malaria and its control measures and attitudes towards community involvement among female community health volunteers in a rural community in Ebonyi State. It was part of a larger study on the implementation of a community-based intervention on the diagnosis and treatment of malaria in pregnancy and in children under five in the same rural community.

## Methods

### Study area

This study was conducted in Amagu community in Abakaliki Local Government Area (LGA) of Ebonyi State. Amagu community has two political wards- Amagu ward and Amagu Unuhu ward. There are 29 major villages in the community. In Amagu community, the people are mainly farmers. The major language in Ebonyi State is Igbo and the dialect of the Amagu people is Izzi. Amagu has seven government owned health facilities: six primary health care centres and one general hospital. There is also a military clinic and a private clinic.

### Study design and population

We conducted a before and after intervention study among female community volunteers (N=29) who were selected by their village leaders and women group leader. Volunteers who had minimum primary school certificate education, were residing in their villages within Amagu community for at least the past three years, could speak the local dialect fluently, had no previous case of misconduct and were willing to participate in the study were considered eligible. Those who were ill at the time of recruitment or were scheduled to travel out of the village for more than a week at a time were excluded.

### Sampling technique

Multistage sampling technique was used to select the community. Abakaliki LGA was selected for the larger study by simple random sampling by balloting from among the four LGAs in Ebonyi North Senatorial zone. (The other two senatorial zones in the state are Ebonyi Central and Ebonyi South and together have nine LGAs) Among the rural communities in Abakaliki LGA, Amagu Izzi community was selected by simple random sampling using the balloting technique. One female community volunteer per village was selected by the village leaders and women group leaders from the twenty-nine villages in the community.

### Data collection

#### Pre-intervention

A semi structured interviewer administered questionnaire adapted from the Household survey KAP Questionnaire of a study in Swaziland [25] was used to collect data on socio-demographic characteristics, knowledge of malaria (cause, transmission, signs and symptoms, those at risk of contracting it) and its control (preventive measures, antimalarial drugs), perception of community ownership of malaria preventive strategies and previous participation in health service delivery activities.

#### Study intervention

The intervention consisted of training sessions on knowledge of malaria and its control as well as conduction of supportive supervision and mentorship for the community health volunteers. They were also trained to give health education on malaria prevention and refer community members with suspected malaria in pregnancy and malaria in children under five to the public health facilities in the community. The community volunteers had four training sessions, one session a day for two hours from May to June 2019. The first training session was conducted on the day of the pretest immediately after the pretest, the second training session, three days later while two refresher trainings were given - four weeks from the first training and a further four weeks later. The training comprised knowledge of malaria ,prevention and treatment giving health education on malaria preventive practices including the correct usage of ITN, interviewing techniques, technique of identification of a raised temperature above normal using a thermometer, identification of signs and symptoms of malaria, documentation in their notebooks and referral of pregnant women and caregivers of children under five years with suspected malaria to the public health facilities in the community. They were taught to also counsel pregnant women on the importance of appropriate ANC attendance and caregivers of children under five years to ensure the children receive complete immunization. The researchers assisted by one of the Officers in charge (OICs) who was the assistant malaria focal person for the LGA and research assistants served as the facilitators using a training manual adapted from one for Community Health Extension Workers (CHEW) and Community Resource Persons developed by The Federal Ministry of Health National Malaria Control Programme (Training Manuals on Malaria Service Delivery Module 5: Malaria in Pregnancy) as well as flipcharts and posters. Interactive lectures, role plays and practical demonstrations were the methodology used.

**Post intervention:** we conducted a post test on the community health volunteers six months after the last training session with the same questionnaire used in the pretest.

### Data analysis

The data was analyzed using Epi Info version 7.2.3 (CDC Atlanta). The questions for knowledge were 5-point Likert like questions which sought to ascertain the knowledge of participants. The questions were scored with a minimum of 1 and maximum of 5 for each question. There were two question stems for the knowledge of the appropriate treatment of malaria, 13 stem questions for the knowledge of malaria signs and symptoms, 19 question stems for the knowledge of the malaria preventives measures, and 5 stem questions for the knowledge of at risk group for severe malaria. For overall knowledge, we used the sum of all the 39 questions. Each question had five options, and this ranged from strongly disagree, disagree, neutral / unsure, agree to strongly agree. The least correct option is scored 1 and the most correct is scored 5. Each community volunteer served as their own control. The mean score of the participants were compared in the pre and six months posttest using paired t test at 5% level of significance.

### Ethical consideration

Prior to the commencement of the study, we obtained ethical approval to carry out the study from the Research and Ethics Committee of Ebonyi State University with reference EBSU/DRIC/UREC/Vol 03/047 and ethical approval from the State Ministry of Health with reference SMOH/48/017. Written informed consent was obtained from the participants (Community Health volunteers and CHEW) before the study and information obtained was handled with strict confidentiality.

## Results

### Socio-demographic characteristics of the participants

The mean age of the participants was 28.5 ± 6.0 years. They were all females. Twenty-three of them (79.3%) were married and majority of them (82.8%) had attained secondary education as their highest level of education. Thirteen (44.8%) of them were unemployed as at the time of their recruitment for the community volunteer task (Table 1).

**Table 1:** sociodemographic characteristics of the community volunteers (n = 29)

Variable	Frequency	Percentage
**Mean age (± SD)**	28.5 ± 6.0	
**Age (years)**		
15-19	1	3.5
20-24	4	13.8
25-29	14	48.3
30-34	5	17.2
35-39	3	10.3
≥40	2	6.9
**Marital status**		
Married	23	79.3
Single	6	20.7
**Education (highest level)**		
Secondary	24	82.8
Tertiary	5	17.2
**Religion**		
Christianity	29	100
**Occupation**		
Artisan	9	31.0
Civil servant	6	20.7
Trader	1	3.5
Unemployed	13	44.8

### Knowledge of community volunteer on malaria control

Generally, there was improvement in the knowledge of the community volunteers about the control and management of malaria up to six months after their training. Specifically, there is significant improvement in the mean scores of their knowledge of malaria signs and symptoms (p < 0.001), knowledge of at risk population for malaria (p = 0.014), preventive measures (p < 0.001) and appropriate drug treatment (p < 0.001, Table 2).

**Table 2:** knowledge of malaria control and management by the community volunteers

Variable	Pre test Mean score (SD)	Post test Mean score (SD)	Paired t test	P value
Knowledge of signs and symptom of malaria	52.4 (8.2)	62.6 (3.9)	6.043	<0.001
Knowledge of at-risk population	19.0 (4.4)	22.0 (3.40	2.616	0.0142
Knowledge of preventive measures	68.8 (8.8)	75.3 (4.8)	3.829	0.0007
Knowledge of appropriate drug treatment	7.6 (2.3)	9.8 (0.5)	4.757	0.0001
Overall knowledge	147.8 (17.9)	169.8 (5.3)	6.335	<0.001

Minimum and maximum scores for signs and symptoms (13, 65), at risk population (5, 25), preventive measures (19, 95) appropriate treatment (2, 10) and overall knowledge (39, 195)

### Perception of the community volunteer on malaria control

There was an additional positive improvement in the perception of community volunteers in their belief that community should be involved in promoting referral of pregnant women with fever (p = 0.001), promoting the use of IPTp (p = 0.048), generating funding initiative to sustain malaria preventive activities (p = 0.037). The community volunteers had positive perception about the need for community involvement in prevention of malaria among pregnant women and children under five years generally, and this was not significantly affected by their participation in the project. Similarly, their involvement did not significantly improve their perception about promoting the use of ANC services within (p = 0.133) and outside the community (p = 0.059), use of LLIN (p = 0.086), environmental sanitation (p = 0.406) and cooperating with agencies and projects that promote preventive practices for malaria in pregnancy (p = 0.281; Table 3). Post intervention, there was significant improvement in the perception of the need for their role in referring pregnant women with fever to health facilities using history, signs and symptoms (p = 0.046), their support for the community to sustain the program (p = 0.031) and for community directed distribution of SP to pregnant women at home (p = 0.033). The intervention did not affect their perception that their role would lead to positive outcomes (Table 4).

**Table 3:** perception of community volunteers on need for community involvement in preventive practices for malaria

Variable	Pre test mean score (SD)	Post test mean score (SD)	Paired t test	P value
Community should be involved in the prevention of malaria among pregnant women and under five children	4.0 (1.6)	4.4 (1.2)	0.812	0.424
Community involvement in promoting use of ANC in the community	4.6 (0.8)	4.8 (0.4)	1.548	0.133
Community involvement in promoting use of ANC outside the community	3.7 (1.4)	4.3 (1.2)	1.967	0.059
Community involvement in promoting the referral pregnant women with fever to health facility by trained community volunteer	4.2 (1.1)	4.9 (0.3)	3.357	0.0023
Community involvement in promoting the referral pregnant women with fever to health facility by members of the community	4.0 (1.1)	4.8 (0.4)	3.633	0.0011
Community involvement in promoting the use of IPTp by pregnant women	4.2 (1.3)	4.8 (0.8)	2.071	0.048
Community involvement in promoting the use of LLIN by pregnant women	4.6 (0.9)	4.9 (0.4)	1.778	0.086
Community involvement in promoting clean environment to discourage the breeding of mosquito	4.4 (0.9)	4.7 (0.9)	0.844	0.406
Community involvement in cooperating with agencies and projects that promote prevention of malaria in pregnancy	4.6 (0.8)	4.8 (0.5)	1.099	0.281
Community involvement in generating funding initiatives to sustain malaria preventive activities after the project ends and agencies leave	4.4 (0.8)	4.8 (0.5)	2.188	0.037

Minimum possible score = 1 and maximum possible score =5

**Table 4:** perception of the community volunteers on anticipated positive outcomes of their role, need for community sustainability and their acceptance of community directed distribution of SP

Variable	Pre test mean score (SD)	Post test mean score (SD)	Paired t test	P value
Having trained community volunteers refer pregnant women with fever to health facilities using history, signs and symptoms is acceptable to me	4.6 (0.9)	4.9 (0.3)	2.087	0.046
Having trained community volunteer refer pregnant women with fever to health facility will improve treatment and encourage health facility utilization	4.6 (0.7)	4.9 (0.4)	1.565	0.129
I will support the community in sustaining this	4.4 (0.9)	4.9 (0.4)	2.268	0.031
I will support the community directed distribution of SP to pregnant women at home if such initiative is brought to my community	4.3 (1.1)	4.9 (0.4)	2.241	0.033

Minimum possible score = 1 and maximum possible score = 5

## Discussion

The aim of this paper was to determine the effect of a capacity building intervention on malaria knowledge, control measures and attitudes towards community involvement among female community health volunteers. It is interesting to note the high knowledge scores (above average. Average referring to half of the maximum obtainable score) pre intervention. A possible explanation is the long interaction of the MAPS program with this community - four years from 2011 to 2015 during which they undertook net distribution campaigns, routine net distribution, health education and sensitization on use of the health facilities. Since the community health volunteers in this study all had minimum secondary education, their understanding was above average. The significant increase in knowledge scores post intervention is similar to the findings of a training on Knowledge, Attitude and Practices of Malaria Prevention and Control among Community Role Model Care Givers in South Western Nigeria [26] where the average knowledge score of cause, transmission, risk factors and consequences, awareness of common symptoms and preventive practices improved post-training .The results are also in agreement with those of a capacity building program for community leaders in Vietnam [27] and the training of village health volunteers on knowledge of dementia [28].

Training has also been shown to increase the role performance of community health volunteers [29]. The significant improvement in the perception of community volunteers in their belief that community should be involved in promoting referral of pregnant women with fever, promoting the use of IPTp, and generating funding initiative to sustain malaria preventive activities are likely to be attributed to fact that some of these were activities undertaken by the community health volunteers in their community engagement with pregnant women and caregivers of under five children in house to house visits between their pre-test ( before the training and supervision) and post-test. Having thus interfaced with the target population, the community volunteers could more easily appreciate the specific need for community involvement in these activities whereas outcomes like their perception about promoting the use of ANC services and use of LLIN were not significantly changed in the post test due to fact that these more basic messages were given during the net distribution campaigns. It is worthy of note that there was a significantly higher perception of the community volunteer support of community sustainability of the programme. Sustainability of the programme would help in driving the intended impact which includes reducing morbidity and mortality due to malaria in pregnancy and childhood. This study, despite its limitations of not having a control group, provides information on the importance of capacity building of community health volunteers in strengthening service delivery.

## Conclusion

We recommend that the stakeholders in malaria control should consider factors that would lead to the success and sustainability of a community health volunteer strategy for the control of malaria in pregnancy and children under five years by health promotion activities during community engagement. This can be subsequently incorporated into existing policies on malaria control in resource constrained environments like ours.

### What is known about this topic

- Community health workers help link the population with health institutions;
- Community health workers have played vital roles in several health interventions.

### What this study adds

- Improving the capacity of the community health workers enhanced their perception of community involvement in promoting referral of cases of fever;
- However, building their capacity did not significantly increase their perception of community involvement in promoting the use of ANC services and the use of LLIN.
